# Lung fibroblasts from patients with emphysema show markers of senescence *in vitro*

**DOI:** 10.1186/1465-9921-7-32

**Published:** 2006-02-21

**Authors:** K-C Müller, L Welker, K Paasch, B Feindt, VJ Erpenbeck, JM Hohlfeld, N Krug, M Nakashima, D Branscheid, H Magnussen, RA Jörres, O Holz

**Affiliations:** 1 Hospital Großhansdorf, Center for Pneumology and Thoracic Surgery, D-22927 Großhansdorf, Germany; 2 University of Lüneburg, Institute of Environmental Chemistry, D-21335 Lüneburg, Germany; 3 Fraunhofer Institute of Toxicology and Experimental Medicine, Department for Clinical Inhalation, D-30625 Hannover, Germany; 4 Institute and Outpatient Clinic for Occupational and Environmental Medicine, Ludwig-Maximilians-University, D-80336 Munich, Germany

## Abstract

**Background:**

The loss of alveolar walls is a hallmark of emphysema. As fibroblasts play an important role in the maintenance of alveolar structure, a change in fibroblast phenotype could be involved in the pathogenesis of this disease. In a previous study we found a reduced *in vitro *proliferation rate and number of population doublings of parenchymal lung fibroblasts from patients with emphysema and we hypothesized that these findings could be related to a premature cellular aging of these cells. In this study, we therefore compared cellular senescence markers and expression of respective genes between lung fibroblasts from patients with emphysema and control patients without COPD.

**Methods:**

Primary lung fibroblasts were obtained from 13 patients with moderate to severe lung emphysema (E) and 15 controls (C) undergoing surgery for lung tumor resection or volume reduction (n = 2). Fibroblasts (8E/9C) were stained for senescence-associated β-galactosidase (SA-β-Gal). In independent cultures, DNA from lung fibroblasts (7E/8C) was assessed for mean telomere length. Two exploratory 12 k cDNA microarrays were used to assess gene expression in pooled fibroblasts (3E/3C). Subsequently, expression of selected genes was evaluated by quantitative PCR (qPCR) in fibroblasts of individual patients (10E/9C) and protein concentration was analyzed in the cell culture supernatant.

**Results:**

The median (quartiles) percentage of fibroblasts positive for SA-β-Gal was 4.4 (3.2;4.7) % in controls and 16.0 (10.0;24.8) % in emphysema (p = 0.001), while telomere length was not different. Among the candidates for differentially expressed genes in the array (factor ≥ 3), 15 were upregulated and 121 downregulated in emphysema. qPCR confirmed the upregulation of insulin-like growth factor-binding protein (IGFBP)-3 and IGFBP-rP1 (p = 0.029, p = 0.0002), while expression of IGFBP-5, -rP2 (CTGF), -rP4 (Cyr61), FOSL1, LOXL2, OAZ1 and CDK4 was not different between groups. In line with the gene expression we found increased cell culture supernatant concentrations of IGFBP-3 (p = 0.006) in emphysema.

**Conclusion:**

These data support the hypothesis that premature aging of lung fibroblasts occurs in emphysema, via a telomere-independent mechanism. The upregulation of the senescence-associated IGFBP-3 and -rP1 in emphysema suggests that inhibition of the action of insulin and insulin-like growth factors could be involved in the reduced *in vitro*-proliferation rate.

## Background

Lung fibroblasts from patients with emphysema show a reduced proliferation rate [1,2], altered growth factor response [3] and lower number of population doublings in long-term culture [1]. Together with clinical observations, these findings lend support to the hypothesis that premature aging of structural cells is involved in the pathogenesis of emphysema. Senescent cells not only loose their ability to divide and respond to mitogenic stimuli but also display alterations in morphology and metabolic profile [4]. This phenotype can be induced by oxidative stress [5], in association with epigenetic changes in gene expression [6,7]. As fibroblasts provide part of the lung's structural support and matrix that is essential for its integrity [8], a senescent phenotype could affect tissue microbalance and structural maintenance of the lung. We thus focused on lung fibroblasts as important players, keeping in mind that it is unlikely that alterations found in these cells are strictly limited to this type of structural cell.

One well-known marker of cellular senescence is senescence-associated β-galactosidase (SA-β-Gal) [9,10]. Its expression depends on confluence [11] and aged cells are positive for SA-β-Gal most likely due to an increased lysosomal content [10].

Among the mechanisms implicated in cellular aging, the telomere hypothesis [12] is based on the fact that telomere length is reduced in each cell division. A length below a critical value induces cell cycle exit and thereby limits the cell's replicative capacity. Indeed, telomeres shorten during aging of cultured fibroblasts [13] and their initial length correlates with replicative capacity [14]. However, an unaltered telomere length would not disprove the hypothesis of aging, as replicative senescence can also be mediated by telomere-independent mechanisms [4].

To elucidate further potential mechanisms, targets selected from an exploratory 12 k cDNA array analysis were reevaluated by quantitative PCR (qPCR), with emphasis on genes related to proliferation and aging. We focused on insulin-like growth factor-binding proteins (IGFBP), as they might mediate between systemic and local alterations in COPD. IGFBP-3 [15] and IGFBP-related protein (rP)-1 (IGFBP-7) [16,17] are associated with senescence, and IGFBP-5 is involved in regulating lung matrix composition [18] and development [19]. It was found to be downregulated with increasing age [20] but upregulated in whole lung samples from severe emphysema [21]. IGFBP-rP2 (CTGF, connective tissue growth factor) and IGFBP-rP4 (Cyr61, cysteine-rich angiogenic inducer 61) are also of interest in this respect [22]. To cover a broad mechanistic spectrum of further candidates that are known to be implicated in cell cycle regulation or senescence, we selected FOSL1 (fos-like antigen 1, Fra-1), a family member of Fos transcription factors [23], LOXL2 (lysyl oxidase-like 2), a member of the lysyl oxidase (LOX) family [24], OAZ1 (ornithine decarboxylase antizyme 1), an inhibitor of the ornithine decarboxylase [25], and CDK4 (cyclin-dependent kinase 4).

Thus the aim of the present study was to further characterize the phenotype of primary parenchymal lung fibroblasts in emphysema and to obtain further clues regarding the hypothesis that premature cellular aging plays a role in this disease. For this purpose we compared SA-β-Gal activity, telomere length, and the expression of a selected panel of genes between lung fibroblasts from patients with emphysema and control patients.

As a result we found that a higher proportion of fibroblasts from patients with emphysema exhibited SA-β-Gal activity and that these cells showed an increased expression of senecence-associated IGFBP-rP1 and IGFBP-3 genes and of IGFBP-3 protein, whereas no difference in telomere length could be detected compared to fibroblasts from controls.

## Methods

### Patients

Primary lung fibroblasts from 13 patients with moderate to severe lung emphysema and 15 patients without clinical, morphological or functional signs of COPD (control) were included (Table 1). All patients were undergoing surgery for lung tumor resection except for two undergoing volume reduction surgery. All patients were smokers except for two patients without COPD. The diagnosis of emphysema took into account all available information, including patients' history, symptoms, chest X-ray (11C, 10E) or CT (7C, 10E), histology, lung function comprising expiratory flow-volume curves, resistance loops and plethysmographic lung volumes, as well as diffusion capacity for carbon monoxide (3C, 5E). The study was approved by the local Ethics Committee and all patients gave their written informed consent.

**Table 1:** Patients' characteristics (all patients, for data of subgroups see Results)

		Control		Emphysema	
n		15		13	
Age	yr	62	(54; 67)	64	(58; 70)
Sex	m/f	10/5		13/0	
BMI	kg/m^2^	24.9	(24.3; 26.6)	22.4	(22.2; 23.9) *
VC	%pred	101.7	(87.2; 110.5)	73.6	(71.6; 90.0) **
FEV_1_	%pred	94.7	(83.9; 106.3)	37.2	(33.5; 40.7) **
FEV_1_/VC	%pred	99.0	(94.3; 104.2)	53.2	(44.6; 54.0) **
ITGV	%pred	104.0	(90.2; 117.8)	181.0	(167.0; 222.3) **
RV	L	2.12	(1.86; 2.52)	5.44	(4.53; 6.42) **
TLC	L	6.47	(4.60; 7.05)	8.7	(8.02; 9.89) **
RV/TLC	%	38.1	(32.6; 45.1)	64.1	(54.1; 67.8) **
Smoking history	packyears	25	(20;40)	50	(40;70) *
COPD stage	0/I/II/III/VI	15/0/0/0/0		0/0/1/11/1	
DT	h	24.8	(21.7; 25.5)	31.2	(29.3; 40.9) **

The table shows median values and quartiles (in parentheses), *p < 0.05, **p < 0.01 regarding the difference between groups. BMI = body mass index, VC = vital capacity, FEV_1 _= forced expiratory volume in one second, ITGV = intra thoracic gas volume, RV = residual volume, TLC = total lung capacity, DT = doubling time (time needed for doubling of cell number) of parenchymal lung fibroblasts in culture. Predicted values were taken form ERS guidelines [36] stages of COPD according to GOLD guidelines .

### Lung fibroblasts

Only lungs from patients without visible/palpable lung metastases were used. Pleura-free parenchymal specimens were excised after careful macroscopic evaluation from peripheral areas of the lobe as far away from the tumor site as possible. The tissue was immediately transferred into explant culture (Dulbeccos Modified Eagles Medium, 10% fetal calf serum) as described previously [1]. As it was necessary to ensure comparable and low passage numbers, only limited amounts of cells were available in each patient. Therefore the different assays comprised different, though overlapping, subgroups of patients. Proliferation and population doublings (PDL) were measured as previously described [1]. Fibroblasts were transferred to 24-well dishes and cell numbers determined manually after 24, 48, 72 and 96 h, while the maximum PDL was determined after weekly passaging until the harvested cell numbers dropped below the initially seeded number of 100.000.

### Staining for Senescence-associated β-Galactosidase (SA-β-Gal)

A total of 10.000–15.000 fibroblasts were transferred onto glass cover slides (18 mm^2^). After culture for 24 h in 6-well plates under standardized conditions (37°C, 5 % CO_2_), staining for SA-β-Gal activity at pH 6.0 was performed (Cell Signaling Technologies, Beverly, MA, USA). Cells positive for the blue stain were counted under visible light, while counter-staining with DAPI enabled the determination of cell number under UV light. To assess sensitivity, 6 independent primary fibroblast cultures were stained in each of three consecutive passages. The proportion of cells positive for SA-β-Gal showed a median (IQR) increase of 5.5 (16.3) % per passage. Thus all staining experiments were performed in passage 4–5.

### Telomere length – Terminal restriction fragment (TRF) length analysis

Cryopreserved cells were thawed, cultured and harvested in passage 2–3 as previously described [1]. DNA was extracted (DNeasy, Qiagen, Hilden, Germany) and digested using RSA I / Hinf I (TeloTAGGG Telomere Length Assay, Roche, Mannheim, Germany). After electrophoretic separation of fragments in 0.8 % agarose gel and blotting (0.2 μm nitrocellulose, 20 × SSC buffer overnight), a DIG-labeled, telomere-specific probe was hybridized to the membrane, coupled with an anti-DIG-alkaline peroxidase conjugate and visualized by chemiluminescence. Mean TRF length was calculated as the sum over the chemiluminescence intensity at each position of the blot, divided by the sum of ratios of intensity at each position to TRF length at that position.

### Gene expression analysis

For exploratory cDNA array analysis fibroblasts were thawed and cultured up to passage 3. Three fibroblast lines from patients with emphysema with a low proliferation rate and three lines from controls with a high proliferation rate as compared to the mean within their group were selected for this experiment. Cells were harvested, immediately frozen and shipped on dry ice for cDNA array analysis (11.835 genes; Atlas™ Plastic Human 12 k Microarray, 634811, Custom Service, BD Biosciences Clontech, Palo Alto, CA, USA). Fibroblasts of each group were pooled, RNA extracted, its quality confirmed by the Agilent Bioanalyzer™ and radio-labeled cDNA probes were hybridized to one array per group. After global normalization and additional correction for GAPDH and β-actin, gene expression was compared between groups (, GSE 3510).

Expression of selected genes was further analyzed by qPCR in independent cultures. Fibroblasts were thawed, cultured up to passage 3; harvested and stored frozen until RNA isolation (RNeasy, Qiagen). A second dish of each line was cultured without fetal calf serum for 2 days prior to harvesting to obtain culture medium for the analysis of total protein and IGFBP-3 concentrations. RNA was transcribed to cDNA using the Qiagen Omniscript-Kit. One primer (sense or anti-sense) was designed intron-spanning using the Primer3 internet-based program . Primer pairs were checked for specific product length by 2 % agarose gel electrophoresis. Primer sequences are listed in Table 2. cDNA of each individual patient was used for quantification by Lightcycler real-time PCR (LC1.0 or LC2.0, Roche) as published previously [26]. Gene expression was normalized by external calibrators for target and reference, as well as by the individual PBGD (porphobilinogen deaminase) expression using RelQuant software V1.01 (Roche).

**Table 2:** Primer sequences used for qPCR

HUGO ID	5'- sense primer- 3'	5'- antisense primer – 3'	product (bp)	Sequence ID
PBGD [26]	CACACAGCCTACTTTCCAAGC	TTCAATGTTGCCACCACACT	155	NM_000190.2
IGFBP-3	CCTGCCGTAGAGAAATGGAA	GAAGGGCGACACTGCTTT	127	NM_000598
IGFBP-5	GAGCAAGTCAAGATCGAGAGAGA	GAAAGTCCCCGTCAACGTA	463	M65062
IGFBP-rP1	CTGCGAGCAAGGTCCTTCCATA	CAGGTTGTCCCGGTCACCA	184	NM_001553
CTGF [22]	CCTGCAGGCTAGAGAAGCAGA	TGCACTTTTTGCCCTTCTTAATGT	90	NM_001901
Cyr61	ACACCAAGGGGCTGGAATG	TGGGGACACAGAGGAATG	193	AF31385
LOXL2	CGGAGGATGTCGGTGTGGT	GCTTGCGGTAGGTTGAGAGG	150	NM_002318
OAZ1	AGGTGGGCGAGGGAATAG	ATGCGTTTGGCGTCTGTG	150	U09202
FOSL1	CAGGCGGAGACTGACAAA	GGGAAAGGGAGATACAAGGT	217	NM_005438
CDK4	TGCAGTCCACATATGCAACAC	CAGCCCAATCAGGTCAAAG	137	M14505

IGFBP-3 protein was analyzed by ELISA (human IGFBP3 Duoset, R&D Systems, Wiesbaden Germany) and total protein by the BCA method [27].

### Data analysis

Owing to the skewed distribution of most variables, median values and quartiles or interquartile ranges (IQR) were chosen for description. Accordingly, the Mann-Whitney U-test was employed for the comparison of groups and the Spearman rank correlation coefficient for assessing the relationship between variables. P-values of less than 0.05 were considered statistically significant.

## Results

### Senescence-associated β-Galactosidase

The subgroups of patients, in which lung fibroblasts were analyzed for SA-β-Gal differed statistically significantly regarding all indices listed in Table 1, except for BMI, smoking history and age (emphysema: n = 8, median (IQR) age 62 (16) yr, FEV_1 _36 (13) %pred; control: n = 9, age 65 (13) yr, FEV_1 _102 (20) %pred). Median (quartiles) doubling time (DT) in passage 2 was 30.7 (28.4; 36.1) h in emphysema and 24.8 (22.8; 25.8) h in control (p = 0.004). The number of population doublings (PD) after thawing of cells was 1.8 (0.5; 3.2) and 4.2 (2.9; 5.7) (p = 0.020).

**Figure 1:**
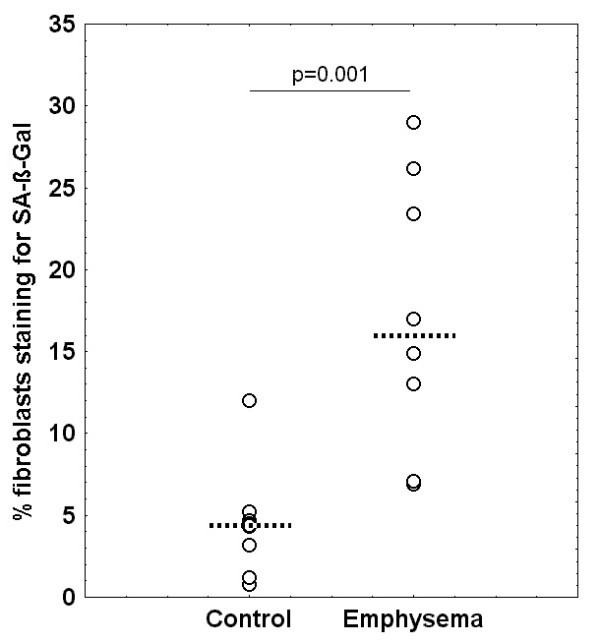
Distribution of the percentage of cells staining positive for SA-β-Gal in control patients and patients with emphysema in passage 4–5 (dotted line: median value).

In emphysema the percentage of fibroblasts staining positive for SA-β-Gal was 16.0 (10.0; 24.8) % compared to 4.4 (3.2; 4.7) % in control samples (p = 0.001, Figure 1). Correspondingly, there was a positive correlation between the proportion of cells positive for SA-β-Gal and DT (r_S _= 0.79, p = 0.0003) and a negative correlation with PD (r_S _= -0.68, p = 0.004).

### Telomere length

The two subgroups of patients whose DNA was analyzed for telomere length differed significantly regarding all indices listed in Table 1, but not for smoking history and age (emphysema: n = 7, age 62 (13) yr, FEV_1 _34 (13) % pred; control: n = 8, age 66 (15) yr, FEV_1 _105 (20) % pred). The median (quartiles) doubling time (DT) in passage 2 was 30.6 (27.4; 33.6) h in emphysema and 24.9 (22.5; 25.6) h in control patients (p = 0.011).

**Figure 2:**
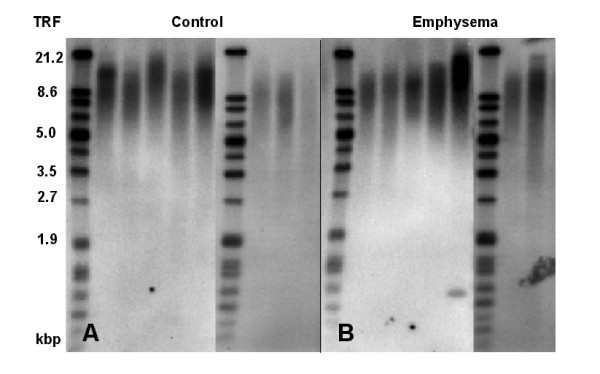
Southern blot of terminal telomere restriction fragments (derived from of Rsa I/Hinf I digestion of DNA samples) detected by chemiluminescence with a DIG-labeled telomeric probe in combination with anti-DIG-alkaline phosphatase (AP) secondary antibody and CDP-star-AP^© ^substrate. Panel A: Samples from 8 control patients (2 separate gels with molecular weight markers). Panel B: Samples from 7 patients with emphysema (2 separate gels with molecular weight markers)

**Figure 3:**
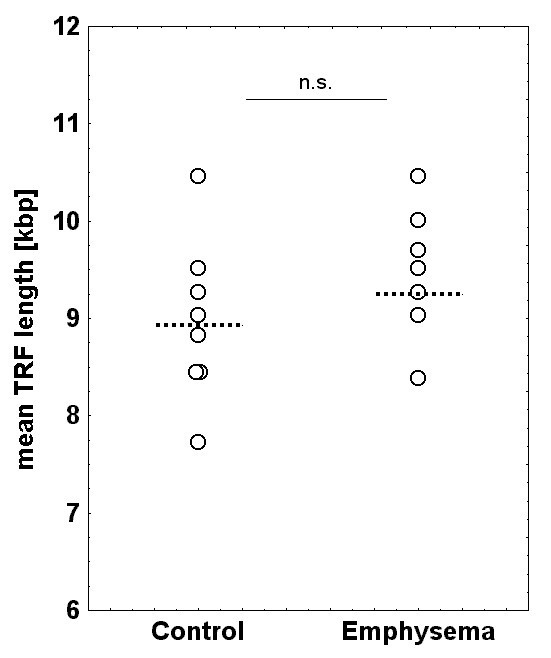
Distribution of mean terminal telomere restriction fragment (TRF) lengths in control patients and patients with emphysema (dotted line: median value).

Terminal restriction fragment (TRF) length did not differ significantly between groups, values being 9.3 (8.6; 10.0) kbp in emphysema and 8.9 (8.3; 9.4) kbp in control (Figures 2 and 3). To assess reproducibility, the assay was repeated in 5 patients per group using the same batch of DNA; the correlation coefficient between these determination was r_S _= 0.75 (p = 0.013).

### Gene expression analysis

For array analysis, fibroblasts of two groups (n = 3 each; emphysema: age 64 (15) yr, FEV_1 _39 (13) %pred; control: age 67 (1) yr, FEV_1 _92 (60) %pred) were used. DT in passage 2 were 40.9, 42.1 and 47.8 h in the individuals with emphysema, and 22.8, 21.2 and 25.5 h in control patients.

There was a factor ≥ 2 difference in expression between groups in 979 genes. To render the conclusions to be drawn for subsequent analysis as safe as possible without missing too many candidates, we then selected genes with a difference of factor ≥ 3, whereby at the same time signal intensities on both arrays were ≥ 2 times the 75-percentile of the intensity distribution of the respective arrays. Fifteen genes were thus found to be upregulated in fibroblasts of emphysema, among them IGFBP-rP1 (4.9-fold), LOX (3.3-fold), LOXL2 (3.9-fold) and TIMP3 (3.0-fold), whereas 121 genes were downregulated, among them CDK4 (6.3-fold), FOSL1 (4.8-fold), OAZ1 (6.7-fold) and IGFBP-5 (5.3-fold).

In order to check these results, gene expression analysis was subsequently performed by qPCR in fibroblasts (passage 2 or 3) of individual patients of two groups of patients (emphysema: n = 10, age 66 (12) yr, FEV_1 _40 (12) %pred; control: n = 9, age 65 (8) yr, FEV_1 _98 (19) %pred). The two groups differed significantly in all variables listed in Table 1, except for BMI and age. The median (quartiles) DT in passage 2 was 31.2 (29.3; 40.9) h in emphysema and 24.8 (21.7; 25.4) h in control patients (p = 0.001).

**Figure 4:**
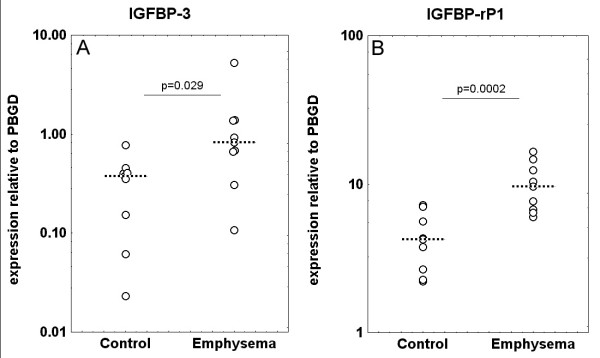
Relative expression of target genes obtained by qPCR in control patients and patients with emphysema. Data points represent normalized ratios of gene expression relative to PBGD and corrected for qPCR calibrators (dotted line: median value). Values are expressed on a log-scale. Panel A: IGFBP-3 expression, Panel B: IGFBP-rP1 expression

No significantly different gene expression was observed by qPCR regarding IGFBP-5, IGFBP-rP2 and -rP4, FOSL1, LOXL2, OAZ1 and CDK4 (Table 3). Regarding IGFBP-3 and IGFBP-rP1, however, expression was significantly higher in emphysema compared to control (Figure 4A/B, Table 3).

**Table 3:** Results of gene expression analysis

Target gene	cDNA array Emphysema/Control	qPCR Controls	qPCR Emphysema	p-value
IGFBP-3	1.3	0.38 (0.15; 0.40)	0.82 (0.66; 1.36)	0.024*
IGFBP-5	0.19	115.0 (60.5; 161.0)	73.6 (49.0; 159.0)	0.51
IGFBP-rP1 (IGFBP-7)	4.87	4.25 (2.67; 5.59)	9.6 (6.77; 12.4)	0.002**
IGFBP-rP2 (CTGF)	n.d.	2.97 (1.68; 4.25)	4.40 (3.86; 4.99)	0.070
IGFBP-rP4 (Cyr61)	0.84	258 (222; 329)	198 (185; 322)	0.57
LOXL2	3.88	1.27 (0.73; 1.79)	1.41 (1.22; 1.92)	0.63
OAZ1	0.15	0.22 (0.17; 0.25)	0.18 (0.18; 0.25)	0.96
FOSL1	0.21	0.89 (0.63; 1.16)	1.00 (0.78; 1.34)	0.57
CDK4	0.16	0.91 (0.58; 1.18)	0.73 (0.53; 0.78)	0.63

The cDNA array column gives the differential gene expression in the exploratory 12 k array experiment as ratio of emphysema to control. qPCR columns give the gene expression of the target gene relative to PBGD for control patients and patients with emphysema. These values are the median values (IQR) of the normalized ratios as derived from RelQuant software. They were additionally corrected by using ratios of qPCR calibrators. P-values refer to the comparison of qPCR results between the two groups and were calculated by the Mann-Whitney U-test. *p < 0.05, **p < 0.01, n.d. not determined.

In culture supernatants collected after 2 days of culture without fetal calf serum, IGFBP-3 was detectable in 8 samples of patients with emphysema and in 9 control samples. After normalizing for the amount of total protein, the median (quartiles) concentration of IGFBP-3 was 1619.6 (1024.1;2937.0) pg/mg protein in emphysema and 505.8 (288.9;779.7) pg/mg protein in controls (p = 0.006).

## Discussion

In the present study we found an increased staining for SA-β-Gal and a qPCR-confirmed upregulation of senescence-associated IGFBP-3 and IGFBP-rP1 in cultured primary parenchymal lung fibroblasts from patients with emphysema; this was supplemented by detection of higher protein levels of IGFBP-3. A comprehensive exploratory microarray analysis suggested that more genes were down- than upregulated in emphysema, though a number of differences could not be confirmed in qPCR. Taken together with the already known reduction in proliferation rate and capacity, these findings provide further evidence for a senescent phenotype of lung fibroblasts in emphysema, in line with the hypothesis, that premature aging of these cells is one of the relevant pathogenetic factors. As mean telomere length was unaltered, the senescent phenotype is more likely to be mediated by telomere-independent mechanisms.

Previous studies already demonstrated that lung fibroblasts from patients with emphysema exhibited a reduced proliferation rate and capacity *in vitro *[1,2]. An increase over time in the proportion of senescent, cell cycle-arrested cells could well be a contributor to tissue destruction. It seems conceivable that such deficiencies favour the onset of emphysematous lesions, and indeed such alterations have been found in senescence-accelerated mice [28]. To check this hypothesis, we first assessed the proportion of cells staining positive for SA-β-Gal, which is considered as a marker of cellular senescence [9]. For this assay we compared the staining between groups after comparable culture times *in vitro*, as a rise in the percentage of SA-β-Gal positive cells can also be observed during aging of cells in culture.

Telomere length, an important marker of cellular aging, which represents a mitotic clock counting down in aging cells, was similar in emphysema and controls. The assay employed is an established procedure and has been successfully used to reveal, for example, shorter telomere lengths in lymphocytes of smokers [29]. The validity of our data was indicated by the similar pattern observed in the duplicate determinations, as well as by the fact that telomere length was close to previously reported values [13]. It might be argued that fibroblasts in emphysema underwent more replications in vivo due to the need for repair of tissue damage and therefore should have shorter telomeres. The characteristics of cell proliferation curves [1] suggest that fibroblasts from emphysema display replicative senescence about 6 population doublings earlier than controls. Assuming a shortening by about 50 bp in each fibroblast replication [13], this difference would correspond to telomeres being about 300 bp shorter in emphysema compared to controls. In opposite to this, mean telomere length as measured in the present study was 400 bp greater. This implies a difference in length of up to 700 bp contra hypothesis which renders it unlikely that shortening of telomeres explained the difference in fibroblast phenotypes. This is true even though the scatter was large and the number of patients investigated was limited. In fact, a statistical analysis showed a less than 5 % probability of obtaining the observed result if the hypothesis of shortened telomeres in emphysema was true. In addition we would like to note that the experiments were performed in early passages. Thus it seems unlikely that the higher in vitro proliferation rate of controls diminished a potential difference to an extent, that it was even reversed into the opposite.

This suggests the presence of telomere-independent replicative senescence which is a well-known phenomenon potentially involving a variety of pathways, including p16 [4,30]. On the basis of this, it does not seem likely that telomere length was the major determinant of the observed alterations in emphysema. It certainly would not explain the differences in proliferation rate, SA-β-Gal staining and gene or protein expression that occurred at comparable telomere lengths.

Two cDNA arrays were used to find hints on differentially expressed genes under baseline culture conditions. mRNA of fibroblasts from patients typical of their group was pooled and analyzed. Based on the results and a comprehensive literature study, the expression of selected genes was then reevaluated in independent cultures from individual patients. As the available cDNA was limited, we focused on a small number of genes associated with senescence and cell cycle, which appeared interesting or novel with regard to the pathogenesis of emphysema. Special attention was paid to using only fibroblasts from cultures with a reproducible proliferation rate to ensure comparability with previous results.

Among the genes that were most upregulated on the array was IGFBP-rP1, whose expression is known to increase during senescence [17]. This family of compounds appeared of particular interest, as it might also provide a bridge between local and systemic effects in COPD via insulin-related pathways, similar to IGFBP-3 and -5. For IGFBP-3 and IGFBP-rP1 the upregulation in emphysema was confirmed by qPCR. Furthermore, increased concentrations of IGFBP-3 were detected in cell culture supernatants of fibroblasts from patients with emphysema. In the qPCR analysis there was also a trend (p = 0.07) towards upregulation of IGFBP-rP2, which had been previously described as overexpressed in lung fibroblasts from emphysema, together with IGFBP-rP4 [22]. We believe that the facts that these authors studied patients with more severe emphysema, as well as differences in methodology are responsible for the differences between the findings.

The upregulation of IGFBP-3 and -rP1 can be taken as further evidence for a senescent phenotype in emphysema. As these proteins interact with mitogenic compounds such as insulin-like growth factor I and II (IGF-I, II) or insulin, an active role for IGFBPs in senescence might well be assumed. Both IGF-I and -II are produced by interstitial mesenchymal cells, epithelial cells and macrophages within the lung, as known from studies in lung fibrosis, and can regulate cell proliferation, especially in fibroblasts [31]. Stimulation of the IGF-I receptor by IGF-I, IGF-II [32] or insulin [33] can promote cell division, possibly in synergy with EGF/EGFR and/or TGF-α [34]. The interaction of IGF-I, -II and insulin with their receptors is largely regulated by IGFBPs and their related proteins [32]. Specifically, elevated mRNA [15,30] or protein levels of IGFBP-3 were found in late passage/senescent fibroblasts [15] and IGFBP-3 is capable of interacting with IGF-I [32]. IGFBP-rP1 can inhibit the growth of cancer cells via a senescence-like mechanism, associated with SA-β-Gal staining [16]. IGFBP-rP1 was also found upregulated in senescent human mammary epithelial cells [17]. Through binding to insulin it can prevent signal transduction towards proliferation. Though the picture regarding the insulin and IGF system is known to be very complex and data are not always consistent, these findings and our results suggest that this system is involved in lung emphysema. It is also important to note that we observed the differences in fibroblast phenotype after several weeks in culture, indicating that these were neither transient nor dependent on the inflammatory environment *in situ*. It does not seem far-fetched to assume the persistence of alterations being at least partially due to epigenetic factors.

In performing the qPCR we additionally covered a number of genes of diverse pathways that could be altered in emphysema or cellular senescence. LOXL2 seemed of interest as involved in cross-linking collagens and elastin [24]; it has been found upregulated in fibroblasts in replicative as well as stress-induced premature senescence [30]. Overproduction of the ornithine decarboxylase (ODC) regulatory protein ODC-antizyme OAZ1 has been shown to correlate with cell growth inhibition in a variety of cell types [25]. This gene was included just because the downregulation in emphysema as indicated by the array would argue against our hypothesis. As a key member of cell cycle-associated factors, CDK4 was included, since there is evidence for a downregulation in senescent cells [35]. In addition, FOSL1 is known to be involved in proliferation and can be upregulated by cigarette smoke [23]. None of these genes turned out to be differentially regulated between emphysema and control patients according to qPCR. This does not render them irrelevant but puts additional emphasis on the findings regarding IGFBP-rP1 and -3, which showed reproducible and meaningful differences between groups. In addition, IGFBP-3 levels were elevated in supernatants of fibroblast from emphysema. These experiments were performed in the absence of fetal calf serum to avoid contributions from the serum. Although serum starvation itself could increase the amount of IGFBP-3 [15], the fact remains that this would have affected both groups. Due to the larger proliferation rate of control fibroblasts a higher total protein concentration was present in the supernatant. To reveal the relative production of IGFBP-3 we therefore normalized to total protein levels.

Due to the limited amount of cells available, it was not possible to perform all investigations in fibroblasts from the same group of patients. We ensured, however, that the groups compared were adequate in each case, by showing that they differed not only with respect to key patients' characteristics but also in fibroblast proliferation rates, as shown previously [1]. The use of different independent cultures, especially for gene expression analysis, thus involved true replicate culture, not just replicate analysis of the same RNA sample. This might well be the cause for the differences between the findings of the exploratory microarray analysis and the qPCR. On the other hand, the fact that IGFBP-3 and -rP1 were upregulated in both analyses and independent cultures, probably gives additional weight to this result.

It has been suggested, that replicative senescence of diploid cells in culture could be due to inadequate growth conditions [5]. Taking into account this, it could be argued, that our observations were at least partially the result of differences in the ability to handle oxidative stress *in vitro*. To resolve this issue, it would be helpful to detect senescence markers in fibroblasts of histological samples. Such analyses are, however, severely handicapped by the lack of fibroblast-specific antibodies. In addition, functional analyses are not possible in these cells without growing them in culture, and single-cell PCR requires amplification of mRNA which is an additional source of error. Thus we infer that, even if cell culture conditions should have been involved in our study, the present data provide evidence that a different phenotype of fibroblasts exists in lung emphysema. Such a different phenotype might well be present in other cells types, too, and is likely to involve epigenetic alterations. The presence of such persistent, programmed alterations might be of considerable importance for all attempts directed towards alveolar regeneration in patients with lung emphysema.

## Conclusion

In conclusion, our data support the view that primary parenchymal lung fibroblasts from patients with emphysema show a senescent phenotype, which does not seem to be based on a reduction of telomere length. Instead, the upregulation of the senescence-associated IGFBP-3 and IGFBP-rP1 suggests that a change in the response to mitogenic and metabolic stimuli such as IGF-I, -II and insulin is involved in the previously found reduced proliferation rate in culture.

## Competing interests

The interpretation and presentation of these results does not influence the personal or financial relationship of any of the authors with other people or organisations.

## Authors' contributions

This work is part of the PhD thesis of KCM, who performed the qPCR analysis, the determination of telomere length and participated in the interpretation of microarray data as well as the preparation of the manuscript. LW performed the macroscopic tissue evaluation, tissue extractions and pathological categorizations. KP did the cell culture, proliferation assays and harvesting of the cells for the different experiments. BF performed the SA-β-Gal experiments and analysis, and participated in cell culture, RNA isolation and cDNA transcription. VJE helped to set up the qPCR, participated in the interpretation of qPCR results and helped with all PCR-related problems. JMH and NK both participated in critically discussing and revising the manuscript and the overall approach. MK and DB selected the patients for this study and participated in the clinical characterization of patients as well as in obtaining informed consent. HM provided the funding of the study and participated in the preparation of the manuscript. RAJ participated in designing the study, the analysis and interpretation of the microarray data and overall results, revised the statistical analysis and took part in writing the manuscript. OH coordinated and critically supervised all experiments, participated in the design of the study and data analysis, and took part in writing the manuscript.
